# A Low Driving-Voltage Hybrid-Electrolyte Electrochromic Window with Only Ferreous Redox Couples

**DOI:** 10.3390/nano13010213

**Published:** 2023-01-03

**Authors:** Jisheng Song, Bingkun Huang, Yinyingjie Xu, Kunjie Yang, Yingfan Li, Yuqi Mu, Lingyu Du, Shan Yun, Litao Kang

**Affiliations:** 1 College of Environment and Materials Engineering, Yantai University, Yantai 264005, China; 2 School of Materials Science and Engineering, University of Science and Technology, Beijing 100083, China; 3 Key Laboratory for Palygorskite Science and Applied Technology of Jiangsu Province, Huaiyin Institute of Technology, Huai’an 223003, China

**Keywords:** electrochromic, smart window, Prussian blue, [Fe(CN)_6_]^4−^/[Fe(CN)_6_]^3−^ redox couple, perfluorosulfonic acid ion-exchange membrane

## Abstract

Even after decades of development, the widespread application of electrochromic windows (ECW) is still seriously restricted by their high price and inadequate performance associated with structural/fabrication complexity and electrochemical instability. Herein, a simple hybrid electrochromic system based on PFSA (perfluorosulfonic acid)-coated Prussian blue (PB, Fe_4_^III^ [Fe^II^(CN)_6_]_3_) film and Ferricyanide–Ferrocyanide ([Fe(CN)_6_]^4−^/[Fe(CN)_6_]^3−^)-containing hybrid electrolyte is reported. The PB film and the [Fe(CN)_6_]^4−^/[Fe(CN)_6_]^3−^ couple show near redox potentials well inside the electrochemical window of water, resulting in a low driven voltage (0.4 V for coloring and −0.6 V for bleaching) and a relatively long lifespan (300 cycles with 76.9% transmittance contrast retained). The PFSA layer, as a cation-exchange structure, significantly improves the transmittance modulation amplitude (*ΔT*: 23.3% vs. 71.9% at a wavelength of 633 nm) and optical memory abilities (*ΔT* retention: 10.1% vs. 67.0% after 300 s open-circuit rest increases) of the device, by means of preventing the direct contact and charge transfer between the PB film and the [Fe(CN)_6_]^4−^/[Fe(CN)_6_]^3−^ couple. This “hybrid electrolyte + electron barrier layer” design provides an effective way for the construction of simple structured electrochromic devices.

## 1. Introduction

As an important optical functional device, electrochromic windows (ECWs) can reversibly change their optical properties (e.g., absorbance, transmittance, and reflectance) under the stimuli of external voltages [1,2,3,4]. Except for the color-switching processes, a well-designed ECW consumes almost no energy to maintain a fixed optical state, demonstrating a desirable optical memory ability [5]. Used as glass facades, ECWs can flexibly adjust the amount of sunlight entering buildings according to ambient temperature and solar irradiance, intelligently improving indoor comfort while minimizing energy consumption [6,7]. In addition to glass facades, these devices may also find applications in various fields including information displays [8,9], anti-glare eyewear [10], and automatic dimming mirrors [9], to name a few.

Generally, an ECW consists of a five-layer-stacked structure, highly like a thin-film battery [11,12,13,14]. The electrochromic layer, which is deposited on a transparent conductive glass substrate, is used as the working electrode. This layer delivers switchable optical properties through an electrochromic reaction. To compensate for the ion/charge fluctuation caused by the electrochromic reaction, the device needs an electroactive ion-storage layer as a counter electrode. The electrochromic and ion-storage layers are ionically conducted by an electrolyte layer. During color switching, the ion-storage layer should dim and bleach simultaneously with the electrochromic layer [15] or always remain highly transparent [16]. In particular, a transparent and colorless ion-charge layer can couple with both cathodic and anodic coloration electrochromic layers, resulting in high device design flexibility [17].

This classical five-layer-stacked device, although working as desired, is costly due to the complicated structure and high manufacturing difficulty [18,19]. Incorporating transparent electroactive ion-storage materials into electrolytes is an effective approach to simplify the device structure [20,21,22,23]. In this device, the hybrid electrolyte provides not only ionic conductivity but also an ion/charge compensating capability thanks to the introduction of ion-storage materials [6]. With electrolyte and ion-storage layers in one, this hybrid-electrolyte ECW shows attractive advantages of simple assembly, fewer side reactions, and flexible capacity matching [21,24,25,26]. Nevertheless, the dissolved ion-storage materials in the electrolyte inevitably migrate to the surface of the electrochromic layer, resulting in high leakage current and, thus, poor optical memory performance [17]. Therefore, a constant current or voltage is consistently required to maintain the desired optical state, remarkably increasing the operational energy consumption of these hybrid-electrolyte devices [17,23].

To solve this problem, electron-barring but ionic-conducting structures are introduced to guarantee the occurrence of the electrochromic reaction while suppressing the leakage current. To this end, Georg et al. developed a WO_3_-based electrochromic device with a I^−^/I_3_^−^-containing hybrid electrolyte and a dense Si_3_N_4_ (or SiO_2_) electron-barrier layer [17]. The barrier layer can reduce the leakage current by 90%, thanks to its dense structure and electronic insulation. Similarly, Leftheriotis et al. deposited a dense ZnS electron barrier layer on a WO_3_ layer via electron beam evaporation to restrain the undesired charge transfer between the oxide EC layer and the Co^2+^/Co^3+^ couple in the electrolyte [27].

**Figure 1:**
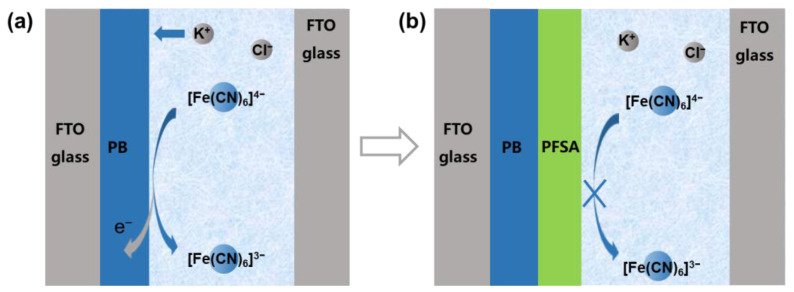
Schematic diagram showing (**a**) the color-fading process of PB-ECW in the open-circuit state and (**b**) the improved optical memory ability of PFSA/PB-ECW by the addition of a PFSA cation-exchange layer.

In this paper, we report a low driving-voltage ECW with a hybrid KCl electrolyte containing a [Fe(CN)_6_]^4−^/[Fe(CN)_6_]^3−^ redox couple, and a PFSA (perfluorosulfonic acid)-coated PB (Fe^III^_4_ [Fe^II^(CN)_6_]_3_) electrochromic electrode. The adoption of the PB-[Fe(CN)_6_]^4−^/[Fe(CN)_6_]^3−^ electrochromic system shows several attractive advantages: (1) the electrochemical reactions of both PB/PW and [Fe(CN)_6_]^4−^/[Fe(CN)_6_]^3−^ couples are ferreous redox processes, which deliver similar redox potentials that can minimize the deriving voltage of the device (0.4 V for coloring and 0.6 V for bleaching); (2) the redox potentials of both PB/PW and [Fe(CN)_6_]^4−^/[Fe(CN)_6_]^3−^ couples are well inside the electrochemical window of water, favoring the exclusion of undesired oxygen/hydrogen evolution reactions; (3) both PB and K_4_ [Fe(CN)_6_]/K_3_[Fe(CN)_6_] are common chemicals that are stable, abundant, and low cost; (4) the [Fe(CN)_6_]^4−^/[Fe(CN)_6_]^3−^ redox couple is highly transparent in the visible and near infrared regions, and thus can match with both cathodic and anodic electrochromic electrodes. However, this hybrid-electrolyte device has very little optical memory ability, due to the dense charge transfer between the directly contacted PB electrochromic film and the [Fe(CN)_6_]^4−^/[Fe(CN)_6_]^3−^ couple in the electrolyte (Figure 1a).

To suppress the charge transfer (i.e., leakage current) between the PB electrochromic electrode and the [Fe(CN)_6_]^4−^/[Fe(CN)_6_]^3−^ couple in the electrolyte, a cation-exchange PFSA layer was coated on the PB electrode (Figure 1b). The abundant sulfonic acid groups in the side chains of PFSA can effectively isolate the [Fe(CN)_6_]^4−^/[Fe(CN)_6_]^3−^ species from the PB electrode through electrostatic repulsion, while simultaneously ensuring the smooth migration of the indispensable K^+^ ions for the electrochromic reaction [28,29] Thanks to the merits of the PFSA cation-exchange layer, the transmission modulation amplitude (*ΔT*) of this hybrid device was increased from 23.3% to 71.9% at 633 nm, and the optical memory ability was increased from 10.1% to 67.0% (i.e., *ΔT* retention) after 300 s open-circuit rest. This “hybrid electrolyte + electron barrier layer” design provides an effective way to construct simple-structured electrochromic devices.

## 2. Experimental Section

### 2.1. Preparation of PB/FTO and PFSA/PB/FTO

The PB layer was deposited onto transparent conducting FTO glass (~10 Ω/□, F-doped SnO_2_-coated glass (Jingjiexin Glass Co., Ltd., Foshan, China) via an electrochemical deposition method, according to the literature [4]. Before deposition, a 25 × 25 mm^2^ FTO glass was thoroughly washed with deionized water and absolute ethanol, sequentially. The cleaned FTO glass was immersed into a mixed aqueous solution containing 10 mM K_3_[Fe(CN)]_6_ (AR grade, Aladdin Bio-Chem Co., Ltd., Shanghai, China), 10 mM FeCl_3_ (AR grade, Sinopharm Chemical Reagent Co., Ltd., Beijing, China) and 50 mM KCl (AR grade, Shanghai Macklin Biochemical Co., Ltd., Shanghai, China), together with an Ag/AgCl reference electrode and a platinum plate counter electrode. The PB layer was electrodeposited onto the FTO glass under a constant current density of −50 mA cm^−2^ for 300 s in this three-electrode configuration. Afterward, the PB/FTO was rinsed with deionized water and dried in air.

A PFSA resin dispersion (5.24 wt%) was purchased from Dongyue Group Co., Ltd., Zibo, China. A 120 μL sample of the dispersion was spin-coated onto the surface of PB/FTO at 500 rpm for 9 s and then 1200 rpm for 30 s (Institute of Microelectronics, Chinese Academy of Sciences, KW-4A Spin Coater). Subsequently, the PFSA/PB/FTO was dried at room temperature for 30 min.

### 2.2. Assembling of PB-ECW and PFSA/PB-ECW

The PB-ECW were assembled with a PB/FTO working electrode and redox electrolyte, which include 0.1 M KCl (AR grade, Shanghai Macklin Biochemical Co., Ltd.), 10 mM K_3_[Fe(CN)_6_] (AR grade, Aladdin Bio-Chem Co., Ltd., Shanghai, China) and 10 mM K_4_ [Fe(CN)_6_] (AR grade, Macklin Biochemical Co., Ltd., Shanghai, China). Firstly, a PB/FTO glass and a bare FTO glass were adhered into a box-like EC device with 1 mm thick pieces of double-sided tape. Afterward, the redox electrolyte was injected into the inner space of the box-like EC device. Finally, the injection hole was sealed with commercial soft adhesives. PFSA/PB-ECW was assembled in the same way as PB-ECW with only the replacement of PB/FTO by PFSA/PB/FTO.

### 2.3. Material Characterization

Surface and cross-sectional morphologies of the FTO glass substrate and PB/FTO glass were observed by a JEOL JSM-7610F field emission scanning electron microscope (SEM). X-ray diffraction (XRD) patterns of the FTO glasses before and after PB-film deposition were collected on a D/max-2500/PC X-ray diffractometer with Cu Kα radiation (λ = 0.1542 nm). Raman spectra were collected on a Lab RAM HR Evolution Raman spectroscope using 785 nm wavelength excitation.

The electrochemical properties of the PB/FTO and [Fe(CN)_6_]^4−^/[Fe(CN)_6_]^3−^ redox couple were characterized by a CHI660D electrochemical workstation using a three-electrode system, in which the measured redox couples were used as the working electrode and a platinum plate and Ag/AgCl electrode as the counter electrode and the reference electrode, respectively. The electrochromic properties of the PB-ECW and PFSA/PB-ECW were measured by two electrodes. The optical transmittance of the PB/FTO, the [Fe(CN)_6_]^4−^/[Fe(CN)_6_]^3−^-containing electrolyte, and the electrochromic devices were collected on a UV-Vis. spectrophotometer (Persee TU-1810, Beijing General Analytical Instrument Co., Ltd., Beijing, China) in a wavelength range of 400 to 1100 nm. To test the electrochromic performance of the ECW, voltage stimulation was applied to the ECW using an electrochemical workstation and the transmittance changes were monitored simultaneously using the spectrophotometer. All electrochemical and optical measurements were performed at room temperature in air.

## 3. Results and Discussion

**Figure 2:**
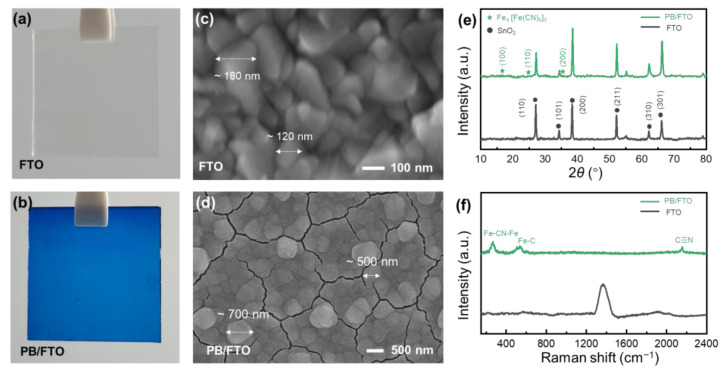
Characterization of FTO and PB/FTO glasses: (**a**,**b**) Digital photographs, (**c**,**d**) SEM images, (**e**) XRD patterns, and (**f**) Raman spectra.

As shown by Figure 2a,b, the colorless FTO glass turns to deep blue after PB deposition, due to the intervalence charge transfer between the cyanide bridged Fe^III^ and Fe^II^ in the PB lattice (Fe^II^-CN-Fe^III^) [30]. Microscopically, the rough FTO film is fully covered by the flat PB layer (Figure 2c,d), in which obvious cracks (80~110 nm in width) stemming from the drying-derived volume changes and internal stress release are detected, a widely reported phenomenon in electrodeposited PB layers (Figure 2d and Figure S1) [31,32]. The PB/FTO glass manifests tiny but sharp XRD peaks at 16.7, 24.8, and 35.3° (Figure 2e) corresponding to the (100), (110), (200) planes of PB (JCPDF card No. 01-0239), respectively, in addition to the strong peaks from FTO glass (SnO_2_, JCPDF card No. 46-1088). In the Raman spectra (Figure 2f), the bare FTO delivers only a broad Raman band at 1300~1500 cm^−1^, while the PB/FTO glass shows three absorption bands at 268, 534, and 2155 cm^−1^, which can be assigned to the deformation vibration of Fe-CN-Fe bonds, the stretched vibration of Fe^III^-N bonds [33,34], and the stretching vibration of C≡N bonds [32,35], respectively. All these characterizations indicate the successful deposition of PB films on the transparent conducting FTO glass.

**Figure 3:**
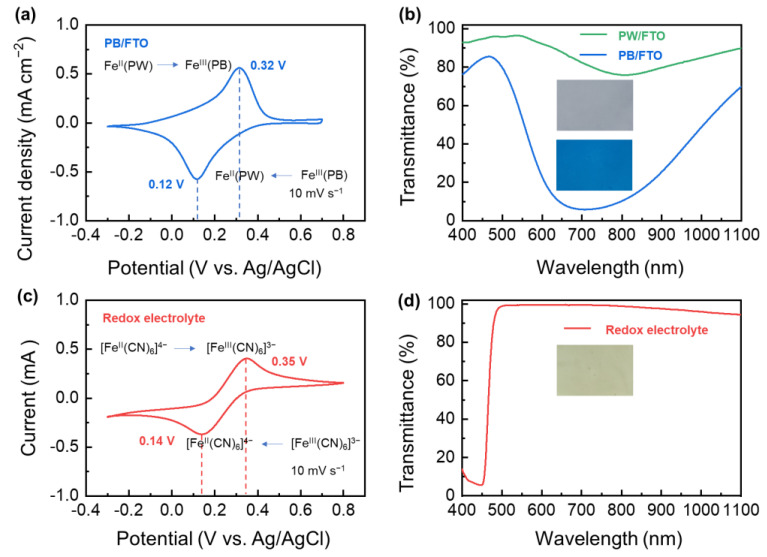
(**a**,**c**) CV curves, (**b**,**d**) transmittance spectra of the PB films (in 0.1 M KCl, **a**,**b**) and redox electrolyte (0.1 M KCl/10 mM K_3_[Fe(CN)_6_]/10 mM K_4_[Fe(CN)_6_], **c**,**d**). The insets in panels (**b**,**d**) show the digital images of corresponding samples.

Figure 3a shows a typical cyclic voltammetric (CV) curve of the PB/FTO glass. A pair of distinct peaks are clearly detected at 0.12 and 0.32 V, corresponding to the cathodic and anodic reactions between PB and PW (Prussian white, K_4_Fe_4_^II^ [Fe^II^(CN)_6_]_3_), as expressed by Equation (1) [36]:

(1) $$\int ∭Fe_{4}^{III}{\left[Fe^{II}{\left(CN\right)}_{6}\right]}_{3}\left(PB,blue\right)+4K^{+}+4e^{-}=K_{4}Fe_{4}^{II}{\left[Fe^{II}{\left(CN\right)}_{6}\right]}_{3}\left(PW,colorless\right)$$

These two peaks show a small polarization voltage (0.2 V) and comparable enclosed areas (12.9 vs. 14.6 mC cm^−2^, Table S1), indicating impressive reaction kinetics and reversibility thanks to the large ionic migration (3.2 Å in the <100> direction) and interstitial spaces (~4.6 Å) in the PB lattice [37]. When K^+^ ions and electrons are co-embedded in the lattice, the Fe^II^-CN-Fe^III^ small polaron is converted to Fe^II^-CN-Fe^II^, leading to the complete disappearance of the intervalence charge transfer absorption band at 600~900 nm and the formation of colorless PW [38]. As a result, the PB coating switches from blue to transparent (Figure 3b). When the inserted K^+^ ions and electrons are de-intercalated from the PW, the film turns back to blue again.

To compensate for the ion/electron fluctuation caused by the above electrochromic reaction, a counter electrode with sufficient charge storage capacity is needed. In this work, we choose the Ferricyanide-Ferrocyanide ([Fe(CN)_6_]^4−^/[Fe(CN)_6_]^3−^) redox couple as the counter electrode material, considering its high transmittance and high solubility in the electrolyte. Figure 3c shows the CV curve of the [Fe(CN)_6_]^4−^/[Fe(CN)_6_]^3−^ redox couple in the KCl aqueous electrolyte, which manifests a cathodic peak at 0.14 V and an anodic peak at 0.35 V. These peaks are at almost the same positions as those of the PB/PW electrochromic electrode since all they are virtually the electrochemical conversion between Fe^II^/Fe^III^. This redox couple also shows a low polarization voltage (0.21 V) and high coulombic efficiency (98.80%, Table S2), suggesting smooth reaction kinetics. In fact, the [Fe(CN)_6_]^4−^/[Fe(CN)_6_]^3−^ redox couple has already been widely used as an electrode in electrochemical characterization [39,40,41], flow batteries [42] and solar cells [43], thanks to its high electrochemical activity and reversibility. At low concentrations, this redox couple is quasi-colorless with high visible and near-infrared transmittance (Figure 3d). Therefore, pairing a PB/PW electrode with the [Fe(CN)_6_]^4−^/[Fe(CN)_6_]^3−^ couple is expected to form an interesting rocking-chair electrochromic system that can operate with small driving voltages.

**Figure 4:**
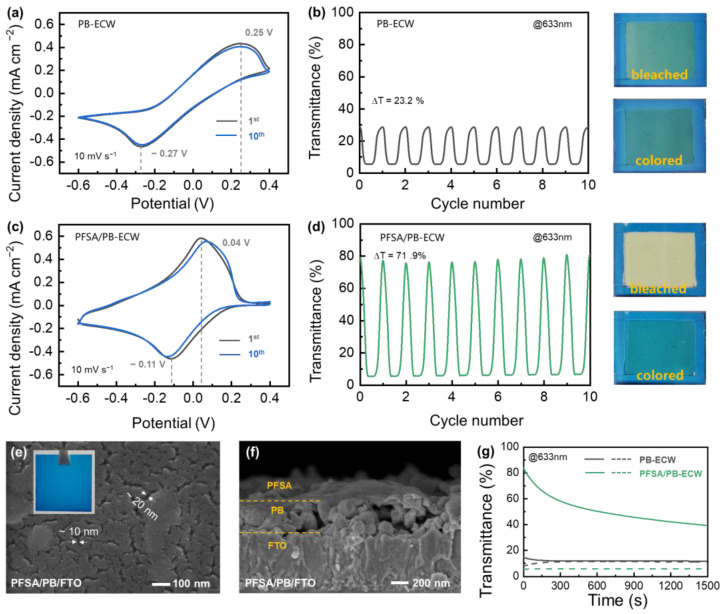
Characterization of the PB-ECW and PFSA/PB-EDW devices: (**a**,**c**) CV curves, (**b**,**d**) transmittance spectra at 633 nm during the CV tests; (**e**,**f**) SEM and optical images of the PFSA/PB/FTO glass; (**g**) optical memory tests in the open-circuit state. The solid and dotted lines are transmittance curves of the corresponding devices in their bleached and colored states, respectively.

Figure 4a shows typical CV curves of the PB-ECW in the [Fe(CN)_6_]^4−^/[Fe(CN)_6_]^3−^-containing hybrid electrolyte (i.e., 0.1 M KCl + 10 mM K_3_[Fe(CN)_6_] + 10 mM K_4_[Fe(CN)_6_]), which display a pair of distinct and stable redox peaks. While requiring low driving voltages (Figure S3, 0.4 V for coloring and −0.6 V for bleaching) compared with typical reported PB-based electrochromic devices (Table S3), this device delivers quite a low transmittance modulation amplitude (23.2% at 633 nm, Figure 4b) due to the dense leakage current as evidenced by the low Coulombic efficiency (65.9%, Table S4). The essence of the leakage current phenomenon is the charge transfer between the directly contacted PB electrochromic layer and [Fe(CN)_6_]^4−^/[Fe(CN)_6_]^3−^ redox couple. To prevent this detrimental charge transfer process while ensuring the indispensable K^+^ migration to complete the electrochromic reactions, spin-coated perfluorosulfonic acid (PFSA) cation-exchange layers were introduced. The abundant sulfonic acid groups on the PFSA chains could effectively insolate the [Fe(CN)_6_]^3−^ and [Fe(CN)_6_]^4−^ from the PB film via electrostatic repulsion [28,44,45]. In addition, the PFSA film possesses high cation conductivity, ensuring fast K^+^ intercalation/de-intercalation kinetics during the electrochromic reaction [46,47]. The beneficial influence of this PFSA layer can be detected by CV and electrochromic tests (Figure 4c,d). In the CV test of PFSA/PB-ECW, the scanning voltage ranges from −0.6 V to 0.4 V at a rate of 10 mV s^−1^ (Figure S2), and the stimulated current density by the voltage variation is shown in Figure 4c. As the voltage decreases from 0.4 V to −0.6 V, the PFSA/PB-ECW switches from blue to transparent. In contrast, when the voltage is increased from −0.6 V to 0.4 V, the PFSA/PB-ECW returns to dark blue. After PFSA coating, the polarization voltage of the PB-ECW is significantly reduced by 70% (from 0.52 to 0.15 V, Table S4), while the Coulomb efficiency and optical modulation amplitude increased from 65.9% to 90.1% (Table S4) and from 23.2% to 71.9% (Figure 4d), respectively, thanks to the suppressed [Fe(CN)_6_]^3−^/[Fe(CN)_6_]^4−^ shuttling.

Figure 4e,f show the SEM and optical images of a PB/FTO glass coated with a PFSA top layer (named PFSA/PB/FTO). The PFSA/PB/FTO glass shows almost the same color as the PB/FTO glass, thanks to the high transmittance of the PFSA layer (Figure S3). Even though the PFSA layer has obvious gullies, it can still improve the optical memory ability of the ECW. As shown in Figure 4g, the PB-ECW exhibits very poor transmittance modulation amplitude and optical memory ability, with both its colored and bleached state completely fading out after only ~200 s. With a PFSA layer to suppress the leakage current, both the colored and bleached states of the PFSA/PB-ECW are remarkably stabilized. After 300 s, and 1500 s rest in an open-circuit state, 67.0% and 42.6% (Table S5) of the optical memory ability remain, respectively, thanks to the excellent cation-exchange ability of the PFSA layer.

**Figure 5:**
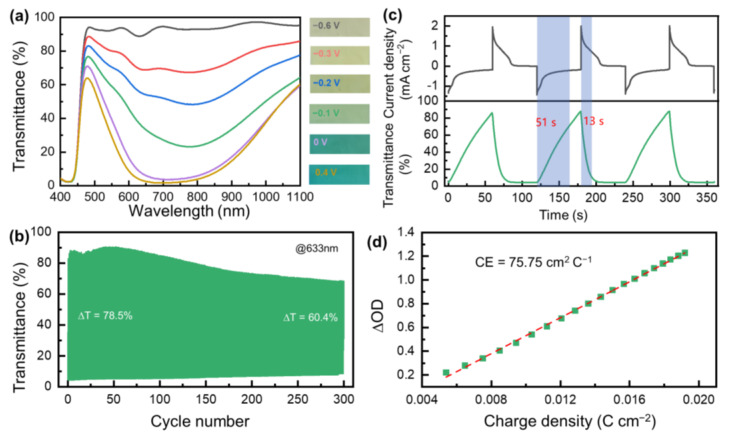
Characterization of a PFSA/PB-ECW device: (**a**) UV–vis transmittance spectra and corresponding digital images at different stimulating voltages; (**b**) Transmittance curves at 633 nm under repeated square-wave potential stimuli; (**c**) Chronoamperometry (CA) curves and corresponding transmittance evolution at the beginning of cycling test; (**d**) Plots of the time-dependent optical density variation as a function of charge density (ΔOD) at 633 nm.

Figure 5a shows the transmittance spectra and optical images of the PFSA/PB-ECW under the stimulation of different voltages. By simply manipulating the voltage, the transmittance of the device can be flexibly adjusted to adapt to frequently changing environments. The colored and bleached states become saturated at −0.6 V and 0.4 V, respectively. This means that with only a low driving voltage (−0.6~0.4 V), the PFSA/PB-ECW achieves an excellent transmittance modulation amplitude of 85.2% (88.8% to 3.6% at 633 nm). Figure 5b further shows the cyclic stability of this PFSA/PB-ECW. Stimulated by a square-wave voltage switching between 0.4 V and −0.6 V with a duration of 60 s (Figure S4), the initial transmittance modulation amplitude of the PFSA/PB-ECW reaches 78.5% (the transmittance modulation is not fully saturated at this condition), comparable to the reported literature (Table S3). The transmittance modulation amplitude decreases to 60.4% after 300 coloring/bleaching cycles. Figure 5c shows the variation in current density and transmittance under square-wave voltage stimulation (Figure S4) at the first 3 cycles, which shows a coloring/bleaching time of 13 and 51 s, respectively [48]. After 300 cycles, the coloring/bleaching times change to 30/49 s (Figure S5). Figure S6 further shows the evolution of the transmittance in a galvanostatic charge/discharge model, which also indicates a low driving voltage. Figure 5d further manifests the optical density-charge density curve for the PFSA/PB-ECW with a low bias voltage (0.4 V). From this curve, the coloration efficiency of the PFSA/PB-ECW is determined to be 75.75 cm^2^ C^−1^ by linearly fitting the slope of this curve [16,49].

## 4. Conclusions

A simple and cheap PB-based ECW hybrid system was designed by adding a [Fe(CN)_6_]^3−^/[Fe(CN)_6_]^4−^ redox couple into the electrolyte. The redox potentials of the [Fe(CN)_6_]^3−^/[Fe(CN)_6_]^4−^ couple and PB film are very near and located within the electrochemical window of water, resulting in a low driving voltage for the ECW (−0.6~0.4 V). In addition, a cation-exchange PFSA layer is coated onto the PB film in order to suppress the charge transfer between the PB film and the [Fe(CN)_6_]^3−^/[Fe(CN)_6_]^4−^ species in the electrolyte. Meanwhile, the cation migration channels in the PFSA layer ensure rapid K^+^ intercalation/de-intercalation and thus fast color switching. On this basis, the optical memory ability and optical modulation amplitude of the PFSA/PB-ECW are significantly improved. A cyclic stability test of the PFSA/PB-ECW shows that 76.9% of the transmittance modulation (at 633 nm) can be retained after 300 cycles. This work provides an alternative solution for the structural design of electrochromic windows.

## Data Availability

Data available on request from the authors.

## Supplementary Materials

The following supporting information can be downloaded at: https://www.mdpi.com/article/10.3390/nano13010213/s1, Figure S1. SEM images of the electrodeposited PB films (50 μA cm^−2^ for 300s) after air-drying. Figure S2. Profile of the cyclic voltametric (CV) test stimulating the bleaching/coloring switching processes of PFSA/PB-ECW. Figure S3. Transmittance spectra of PB/FTO and PFSA/PB/FTO. Figure S4. Profile of the square-wave voltage stimulating the bleaching/coloring switching processes of PFSA/PB-ECW in the chronoamperometry (CA) test. Figure S5. Chronoamperometry (CA) curves and corresponding transmittance evolution at the end of cycling test. Figure S6. galvanostatic charge/discharge (GCD, blue lines) profiles and transmittance variation (green lines). Table S1. Quantitative analyses of the PB film’s initial CV curve. Table S2. Quantitative analyses of the redox electrolyte’s initial CV curve. Table S3. Electrochromic performance comparison of different PB-based devices. Table S4. Quantitative analyses of the initial CV curves of the PB-ECW and PFSA/PB-ECW devices. Table S5. Optical memory abilities of the PB-ECW and PFSA/PB-ECW devices at different open-circuit aging times. References [50,51,52,53] were cited in Supplementary Materials.
