# Supplementary material for: A Low Driving-Voltage Hybrid-Electrolyte Electrochromic Window with Only Ferreous Redox Couples
Source: Nanomaterials (Basel). 2023 Jan 3;13(1):213. doi: 10.3390/nano13010213 (PMC9823981; doi:10.3390/nano13010213)
Supplement: Supplementary file 1 [file nanomaterials-13-00213-s001.zip › nanomaterials-2096424-supplementary.pdf]

nanomaterials

MDPI

Supplementary Materials

A Low Driving-Voltage Hybrid-Electrolyte Electrochromic Window with Only Ferreous Redox Couples

Jisheng Song [1,†], Bingkun Huang [1,†], Yinyingjie Xu [1], Kunjie Yang [1], Yingfan Li [1], Yuqi Mu [2], Lingyu Du [1], Shan Yun [3,*] and Litao Kang [1,*]

1 College of Environment and Materials Engineering, Yantai University, Yantai 264005, China
2 School of Materials Science and Engineering, University of Science and Technology, Beijing 100083, China
3 Key Laboratory for Palygorskite Science and Applied Technology of Jiangsu Province, Huaiyin Institute of Technology, Huai'an 223003, China
* Correspondence: yunshan@hyit.edu.cn (S.Y.); kanglitao@ytu.edu.cn (L.K.)
† These authors contributed equally to this paper and are co-first authors.

Table S1. Quantitative analyses of the PB film's initial CV curve.

Peak location (V)		Capacity (mC cm^{-2})		Initial CEs (%)	Polarization potential (V)
Cathodic	Anodic	Cathodic	Anodic		
0.12	0.32	14.60	12.93	88.56	0.20

Table S2. Quantitative analyses of the redox electrolyte's initial CV curve.

Peak location (V)		Capacity (mC)		Initial CEs (%)	Polarization potential (V)
Cathodic	Anodic	Cathodic	Anodic		
0.14	0.35	18.94	19.17	98.80	0.21

Table S3. Electrochromic performance comparison of different PB-based devices.

Counter electrode	Driving voltage (V)	Coloration efficiency (cm^2 C^{-1}) @ Bias potential (V)	Transmittance modulation (ΔT) @ wavelength	Ref
ZnO	-2.0 ~ 1.3	~	52.0%@686 nm	[1]
Zn	0.5 ~ 1.9	76.8@1.9	84.9%@633 nm	[2]
Zn	0.8 ~ 1.6	131.5@1.6	67.2%@632.8 nm	[3]
Al	0.8 ~ 2.1	~	52.2%@670 nm	[4]
FTO	-1.2 ~ 1.8	87.4@1.8	44.9%@680 nm	[5]
PEDOT-EthC6	-1.4 ~ 1.8	563@1.8	3 ~ 50%@visible light	[6]
Poly(butyl viologen)	-1 ~ 1.7	157@1.7	62.5%@545 nm	[7]
$[Fe(CN)_6]^{3-}$/$[Fe(CN)_6]^{4-}$	-0.6 ~ 0.4	77.75@0.4	78.5%@633 nm*	This work

*A box-like device consisting of two bare FTO glasses and 1mm-thick electrolyte is used as the baseline for transmittance test.

Table S4. Quantitative analyses of the initial CV curves of the PB-ECW and PFSA/PB-ECW devices.

ECW	Peak location (V)		Capacity (mC cm^{-2})		Initial CEs (%)	Polarization potential (V)
	Cathodic	Anodic	Inserted	Extracted		
PB-ECW	-0.27	0.25	27.16	17.89	65.87	0.52

PFSA/PB-ECW	-0.11	0.04	20.72	18.66	90.06	0.15

Table S5. Optical memory abilities of the PB-ECW and PFSA/PB-ECW devices at different open-circuit aging times.

Rest times in open circuit state	Transmittance of PB-ECW			Transmittance of PFSA/PB-ECW		
	Colored	Bleached	*ΔT*	Colored	Bleached	*ΔT*
0 s	7.9	14.8	6.9	5.6	83.9	78.3
300 s	11.2	12.1	0.9	5.7	58.2	52.3
1500 s	11.3	11.7	0.4	5.9	39.3	33.4

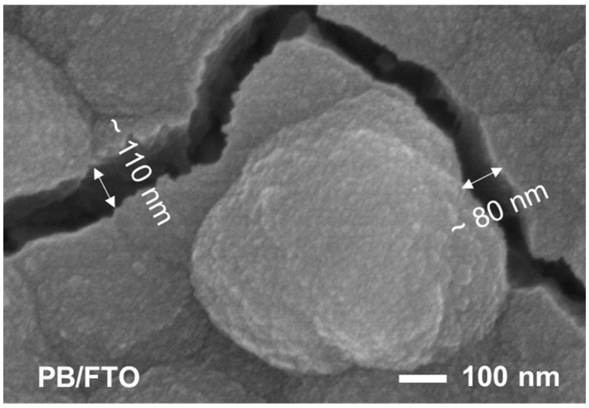

Figure S1. SEM images of the electrodeposited PB films (50 μA cm^{-2} for 300s) after air-drying.

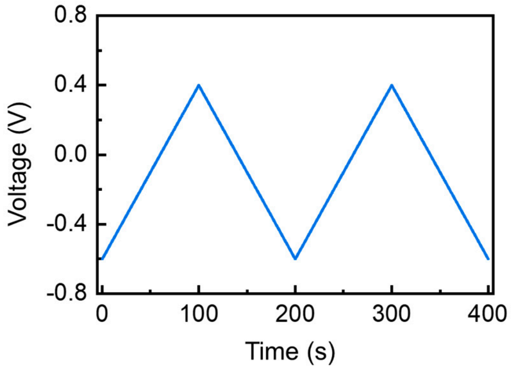

Figure S2. Profile of the cyclic voltametric (CV) test stimulating the bleaching/coloring switching processes of PFSA/PB-ECW.

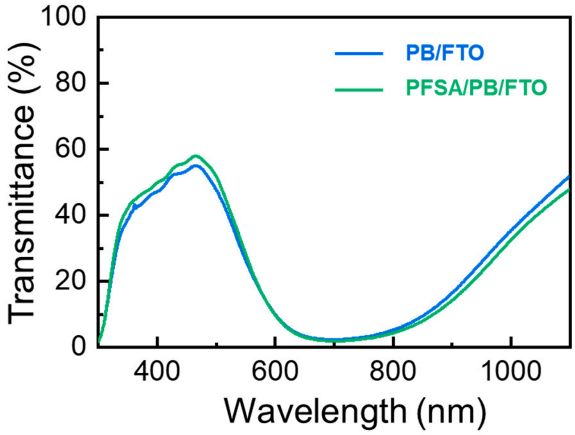

Figure S3. Transmittance spectra of PB/FTO and PFSA/PB/FTO.

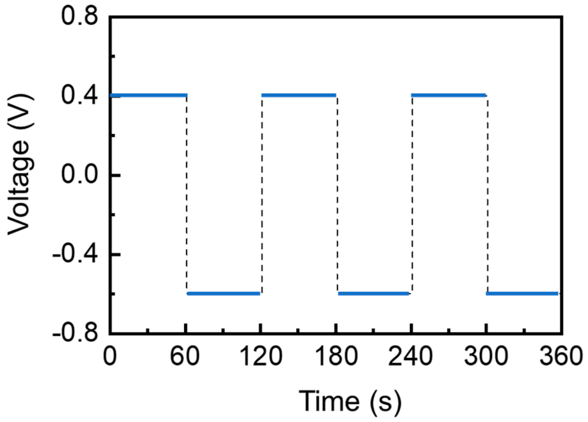

Figure S4. Profile of the square-wave voltage stimulating the bleaching/coloring switching processes of PFSA/PB-ECW in the chronoamperometry (CA) test.

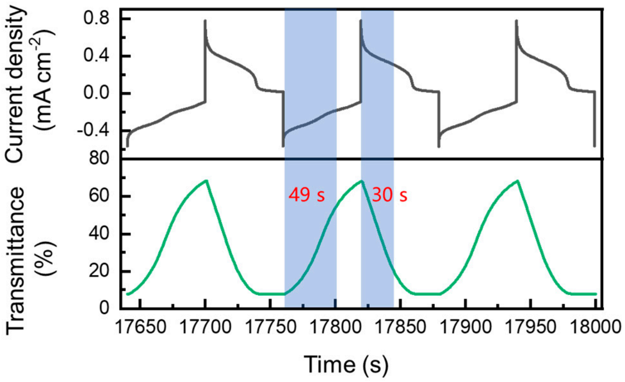

Figure S5. Chronoamperometry (CA) curves and corresponding transmittance evolution at the end of cycling test.

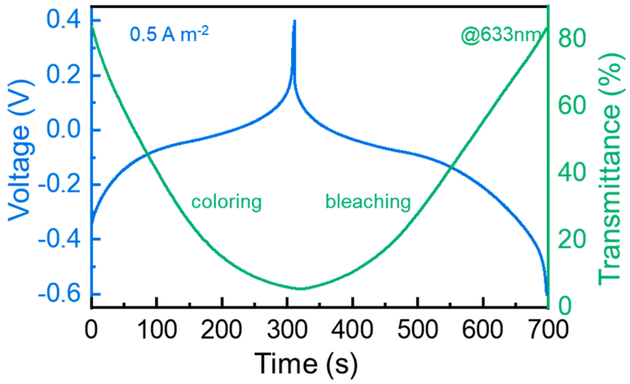

Figure S6. galvanostatic charge/discharge (GCD, blue lines) profiles and transmittance variation (green lines).

References

1. Pham, N.S.; Phan, P.T.Q.; Nguyen, B.N.; Le, V.X. Enhanced stability of electrochromic devices based on Prussian blue by tuning electrolyte ions and charge/discharge balance. *J. Appl. Electrochem.* **2022**, 1–10, https://doi.org/10.1007/s10800-022-01747-1.
2. Wang, B.; Cui, M.; Gao, Y.; Jiang, F.; Du, W.; Gao, F.; Kang, L.; Zhi, C.; Luo, H. A Long-Life Battery-Type Electrochromic Window with Remarkable Energy Storage Ability. *Sol. RRL* **2020**, *4*, 1900425.
3. Li, H.; Zhang, W.; Elezzabi, A.Y. Transparent Zinc-Mesh Electrodes for Solar-Charging Electrochromic Windows. *Adv. Mater.* **2020**, *32*, 2003574. https://doi.org/10.1002/adma.202003574.

4. Ma, D., Eh, A.L.-S., Cao, S., Lee P.S., Wang, J. Wide-Spectrum Modulated Electrochromic Smart Windows Based on MnO_2/PB Films. *ACS Appl. Mater. Interfaces*, **2022**. *14*, 1443–1451.
5. Qian, J.; Ma, D.; Xu, Z.; Li, D.; Wang, J. Electrochromic properties of hydrothermally grown Prussian blue film and device. *Sol. Energy Mater. Sol. Cells* **2018**, *177*, 9–14, https://doi.org/10.1016/j.solmat.2017.08.016.
6. Macher, S., Schott M., Dontigny M., Guerfi A., Zaghib K., Posset U., Löbmann P. Large-Area Electrochromic Devices on Flexible Polymer Substrates with High Optical Contrast and Enhanced Cy-cling Stability. *Adv. Mater. Technol.,* **2020.** *6*, 2000836, https://doi.org/10.1002/admt.202000836.
7. Fan, M.-S.; Kao, S.-Y.; Chang, T.-H.; Vittal, R.; Ho, K.-C. A high contrast solid-state electrochromic device based on nano-structural Prussian blue and poly(butyl viologen) thin films. *Sol. Energy Mater. Sol. Cells* **2016**, *145*, 35–41. https://doi.org/10.1016/j.solmat.2015.06.031.
